# Intensive Versus Non-Intensive Therapy in Older Patients with Acute Myeloid Leukemia: A Real-World Experience

**DOI:** 10.3390/medicina62091733

**Published:** 2026-09-09

**Authors:** Dila Elifsu Yuksekli, Arda Bayar, Ceren Uzunoglu Guren, Fatma Arikan, Fatma Temiz, Ahmet Mert Yanik, Tayfur Toptas, Isik Kaygusuz Atagunduz, Tulin Tuglular, Asu Fergun Yilmaz

**Affiliations:** 1 Department of Medical Oncology, Institute of Oncology, Istanbul University, 34093 Istanbul, Turkey; 2 Department of Hematology, Marmara University Pendik Training and Research Hospital, 34854 Istanbul, Turkey; arda.bayar@me.com (A.B.); cerenuzunoglu92@gmail.com (C.U.G.); fatmagecgelarikan@gmail.com (F.A.); fatma.temiz@gmail.com (F.T.); toptast@gmail.com (T.T.); isikkaygusuz@yahoo.com (I.K.A.); 3 Department of Hematology, University of Health Sciences, Bursa City Training and Research Hospital, 16110 Bursa, Turkey; ahmetmertyanik@gmail.com; 4 Department of Hematology, Acibadem Hospital, 34718 Istanbul, Turkey; ttuglular@gmail.com

**Keywords:** acute myeloid leukemia, 65 years and older, intensive therapy, non-intensive therapy, overall survival

## Abstract

*Background and Objectives*: The optimal treatment strategy for older patients with acute myeloid leukemia (AML) remains uncertain because treatment selection is influenced by age, comorbidity, functional status, and disease biology. This study explored real-world outcomes with intensive and non-intensive treatment strategies. *Materials and Methods*: We retrospectively analyzed 69 patients aged ≥ 65 years diagnosed with AML between 2012 and 2022 at a tertiary center. Treatment intensity was defined by regimen. Baseline demographic, clinical, and laboratory parameters were recorded. Progression-free survival (PFS) and overall survival (OS) were estimated by the Kaplan–Meier method and compared using log-rank tests. *Results*: A total of 69 patients were included, with a median age of 71 years. Intensive and non-intensive therapies were administered in 24.2% and 75.8% of treated patients, respectively. The median PFS was 6.7 months, and the median OS was 7.8 months. One- and two-year OS rates were 29% and 9.2%, respectively. No significant differences in PFS or OS were observed between treatment intensity groups. Among response-evaluable patients, complete remission/complete remission with incomplete hematologic recovery (CR/CRi) was achieved in 58.0% of intensively treated patients and 43.3% of those receiving hypomethylating agent (HMA)-based therapy. Among the evaluated baseline variables, only Eastern Cooperative Oncology Group (ECOG) performance status was significantly associated with overall survival. Patients with an ECOG performance status ≥ 2 had significantly shorter OS than those with ECOG <2 (median OS: 4.85 vs. 8.60 months, *p* = 0.009). *Conclusions*: In this real-world cohort of older patients with AML, no statistically significant differences in PFS or OS were observed between intensive and non-intensive treatment strategies. Although CR/CRi rates were numerically higher with intensive therapy, disease progression remained frequent among patients who achieved a response. ECOG performance status was significantly associated with overall survival, highlighting the relevance of functional status in individualized treatment selection. These findings should not be interpreted as evidence of therapeutic equivalence.

## 1. Introduction

Acute myeloid leukemia (AML) is characterized by clonal proliferation of myeloid blasts in peripheral blood, bone marrow and/or other tissues. It is more common in adults, with a median age of 68 years [1]. Despite advances in treatment, the prognosis of older patients with AML remains poor, with a reported 5-year overall survival (OS) of less than 10% [2]. Comorbidities, along with poorer performance status in this patient group, contribute to a higher risk of treatment-related morbidity and mortality. Moreover, AML in older patients is frequently characterized by biological features associated with chemoresistance, including a higher prevalence of adverse cytogenetic abnormalities, upregulation of multidrug resistance genes such as *MDR1*, and the frequent co-occurrence of antecedent hematologic disorders [3,4]. Consequently, the therapeutic management of older patients with AML remains challenging. Although treatment options have expanded in recent years, selecting the optimal strategy in routine practice remains difficult, and real-world data comparing intensive and non-intensive approaches are limited.

The 7+3 chemotherapy regimen (anthracycline and a continuous infusion of cytarabine) is the standard induction therapy for younger and medically fit patients with newly diagnosed AML; however, this regimen is often not suitable for older patients with AML due to its intensive nature and associated toxicity. For this unfit and older patient population, treatment regimens based primarily on hypomethylating agents (HMAs), with or without the B-cell lymphoma 2 (BCL-2) inhibitor venetoclax, represent an appropriate therapeutic option. These approaches also differ in their response characteristics. Intensive chemotherapy is intended to achieve rapid cytoreduction and complete remission following induction, whereas responses to HMA monotherapy may develop over several treatment cycles; the addition of venetoclax to HMA therapy substantially increases remission rates compared with HMA monotherapy [5,6]. The suitability for intensive therapy in this age group requires individualized decision-making based on age, comorbidities, performance status, and genetic profile through multidisciplinary evaluation.

This study aimed to evaluate the associations of demographic characteristics, laboratory parameters, cytogenetic profiles, and treatment modalities with clinical outcomes and survival in patients aged ≥ 65 years diagnosed with AML at a tertiary university hospital.

## 2. Material and Methods

### 2.1. Patients

The records of all patients diagnosed with AML at the Department of Hematology between 2012 and 2022 were retrospectively reviewed. A total of 100 patients aged ≥ 65 years were identified. Thirty-one patients were excluded because of incomplete clinical or survival data, and 69 patients were included in the final analysis.

Demographic characteristics, comorbidities, Eastern Cooperative Oncology Group (ECOG) performance status, laboratory parameters at the time of diagnosis (complete blood count, creatinine, estimated glomerular filtration rate (eGFR), lactate dehydrogenase (LDH) levels), cytogenetic profiles, and treatment data were retrospectively collected from medical records. Comorbidities were evaluated using the Charlson Comorbidity Index (CCI). eGFR was estimated using the Chronic Kidney Disease Epidemiology Collaboration (CKD-EPI) formula. Renal function was categorized according to eGFR into three groups (≤30, 30–50, and ≥50 mL/min/1.73 m^2^), given the heterogeneity of renal function thresholds reported across AML studies [6].

Patients were categorized into two groups based on the chemotherapy regimen: intensive therapy (7+3 or fludarabine, cytarabine, granulocyte colony-stimulating factor, and idarubicin (FLAG-IDA) induction, followed by high/intermediate-dose cytarabine) and non-intensive therapy (hypomethylating agents [azacitidine or decitabine] with or without Venetoclax).

### 2.2. Treatments

Among the 69 patients included in the study, seven (10.2%) died before treatment initiation and therefore received no antileukemic therapy. Of the remaining 62 patients, 15 (24.2%) received intensive chemotherapy and 47 (75.8%) received non-intensive therapy.

Of the 15 patients receiving intensive therapy, 14 received 7+3 induction chemotherapy and one received FLAG-IDA. The 7+3 regimen consisted of cytarabine administered as a continuous intravenous infusion (200 mg/m^2^/day for 7 days) combined with idarubicin (12 mg/m^2^/day for 3 days). Following induction, patients received high/intermediate-dose cytarabine consolidation. Granulocyte colony-stimulating factor (G-CSF) was not administered routinely. Posaconazole 300 mg orally once daily was used for antifungal prophylaxis. Herpes simplex virus (HSV) serostatus was routinely assessed in all patients before treatment, and antiviral prophylaxis and subsequent infectious-disease management were implemented when indicated according to the recommendations of our institutional Infectious Diseases Committee. Routine Pneumocystis jirovecii pneumonia (PJP) prophylaxis was not used. In four patients with FLT3-positive disease, midostaurin was added to intensive chemotherapy.

Non-intensive treatment was administered to 47 patients and consisted of azacitidine or decitabine, with or without venetoclax. Of these, 41 patients received azacitidine monotherapy, three received decitabine monotherapy, and three received azacitidine plus venetoclax. Azacitidine was administered at 75 mg/m^2^/day subcutaneously or intravenously for 7 consecutive days in a 28-day cycle, whereas decitabine was administered intravenously at 20 mg/m^2^/day for 5 consecutive days in a 28-day cycle. Venetoclax was administered orally with dose adjustment for concomitant posaconazole prophylaxis, using a ramp-up schedule of 20 mg, 50 mg, and 100 mg, followed by a maintenance dose of 100 mg once daily. Patients receiving azacitidine plus venetoclax also received oral posaconazole 300 mg once daily for antifungal prophylaxis. In one patient who developed persistent cytopenia after achieving partial remission, the duration of venetoclax administration was shortened to 14 days per 28-day cycle according to hematologic recovery.

Among patients receiving HMA-based therapy, nearly all were hospitalized during the first treatment cycle to allow close clinical and laboratory monitoring, particularly for tumor lysis syndrome. Subsequent treatment cycles were generally administered and monitored on an outpatient basis when clinically appropriate.

Supportive care, antimicrobial prophylaxis, and transfusion support were provided in both treatment groups according to institutional practice and individual clinical requirements.

### 2.3. Definitions

Treatment response was assessed according to the European LeukemiaNet (ELN) response criteria. Complete remission (CR) was defined as bone marrow blasts < 5%, absence of circulating blasts and blasts with Auer rods, absence of extramedullary disease, an absolute neutrophil count ≥ 1.0 × 10^9^/L, and a platelet count ≥ 100 × 10^9^/L. Complete remission with incomplete hematologic recovery (CRi) was defined as meeting all CR criteria except for residual neutropenia (absolute neutrophil count < 1.0 × 10^9^/L) and/or thrombocytopenia (platelet count < 100 × 10^9^/L). Partial remission (PR) was defined as a decrease in bone marrow blast percentage to 5–25%, with a ≥50% reduction in blast count from baseline, along with the absence of circulating blasts and no extramedullary disease. Refractory disease (RD) was failure to achieve complete remission after two induction chemotherapy cycles or failure to achieve complete remission within 6 months of low-intensity therapy. Relapse was accepted as bone marrow blast count more than 5% after complete remission or peripheral blood blasts that reappeared on two consecutive evaluations, or new extramedullary disease development. Adverse events were reported according to Common Terminology Criteria for Adverse Events (CTCAE).

Cytogenetic and molecular risk stratification was retrospectively performed according to the ELN 2017 classification using the available data. Available variables included FLT3 and NPM1 mutation status, t(8;21), inv(16), and complex karyotype. Extended molecular profiling was available in only two patients.

### 2.4. Survival Analysis

Overall survival (OS) was defined as the time from the date of diagnosis to the date of death, or to the last visit for patients who were still alive. Progression-free survival (PFS) was defined as the time from the date of diagnosis to disease progression, relapse, death from any cause, or the last follow-up, whichever occurred first.

### 2.5. Statistical Analysis

Statistical analyses were performed using SPSS version 25 (SPSS Inc., Chicago, IL, USA). Distributional assumptions for continuous variables were assessed using the Kolmogorov–Smirnov and Shapiro–Wilk tests. Normally distributed variables are presented as mean ± standard deviation, whereas non-normally distributed variables are presented as median (range). Survival functions were estimated using the Kaplan–Meier method and compared using the log-rank test. Survival comparisons were exploratory and unadjusted, and no multivariable or propensity-based adjustment was performed. All tests were two-sided, and *p* < 0.05 was considered statistically significant.

## 3. Results

### 3.1. Patient Characteristics and Laboratory Tests at the Diagnosis

A total of 69 patients aged ≥ 65 years with AML were enrolled in the study. The median age was 71 years (range 65–88), and 55.1% were men. Seventeen patients (24.6%) had secondary AML, of whom 58.9% had transformation from myelodysplastic syndrome (MDS). The median ECOG performance and CCI was 1 (0–3) and 6 (4–9), respectively. The median body mass index (BMI) was 25 kg/m^2^ (range 18–34) and 29.1% were obese. Baseline demographic and clinical characteristics are summarized in Table 1.

The median leukocyte count at the time of diagnosis was 7 × 10^9^/L (range 0.5–293 × 10^9^/L). Fourteen (20.3%) of the patients had leukocytosis above 50 × 10^9^/L and four (5.8%) had signs and symptoms of hyperviscosity and underwent leukapheresis. Baseline laboratory findings are summarized in Table 2.

The 2017 ELN risk classification could be performed in 52 patients (75.4%). Of these patients, 2 (3.8%), 38 (73.1%) and 12 (23.1%) were classified into the favorable-, intermediate-and adverse-risk groups, respectively.

### 3.2. Treatment Outcomes and Survival Analysis

Treatment response was evaluable in 42 of 69 patients (60.8%). Seven patients (10.2%) died before antileukemic treatment could be initiated, and an additional 20 patients (29.0%) died before formal response assessment. Among the 42 response-evaluable patients, 20 (47.6%) achieved CR/CRi, 13 (31.0%) achieved PR, and 9 (21.4%) had refractory disease. During a mean follow-up of 11.44 ± 13.16 months, disease progression was detected in 63.6% of patients who had achieved at least a partial response. CR/CRi rates did not differ significantly according to sex, age, ECOG performance status, CCI, LDH, eGFR, leukocytosis, or cytopenia at diagnosis.

The median PFS was 6.7 months (range, 1–88.5), and 1-year and 2-year PFS rates were 20.3% (95% confidence interval (CI): 12.7–32.4%) and 4.7% (95% CI: 1.6–14.0%), respectively. PFS did not differ significantly according to sex, age, CCI, bone marrow blast percentage, peripheral blood blast percentage, leukocytosis or cytopenia, LDH level, eGFR, BMI or ELN classification. Patients with an ECOG performance status ≥ 2 had a shorter median PFS than those with ECOG <2 (4.85 vs. 7.5 months), although this difference did not reach statistical significance (*p* = 0.052).

**Figure 1:**
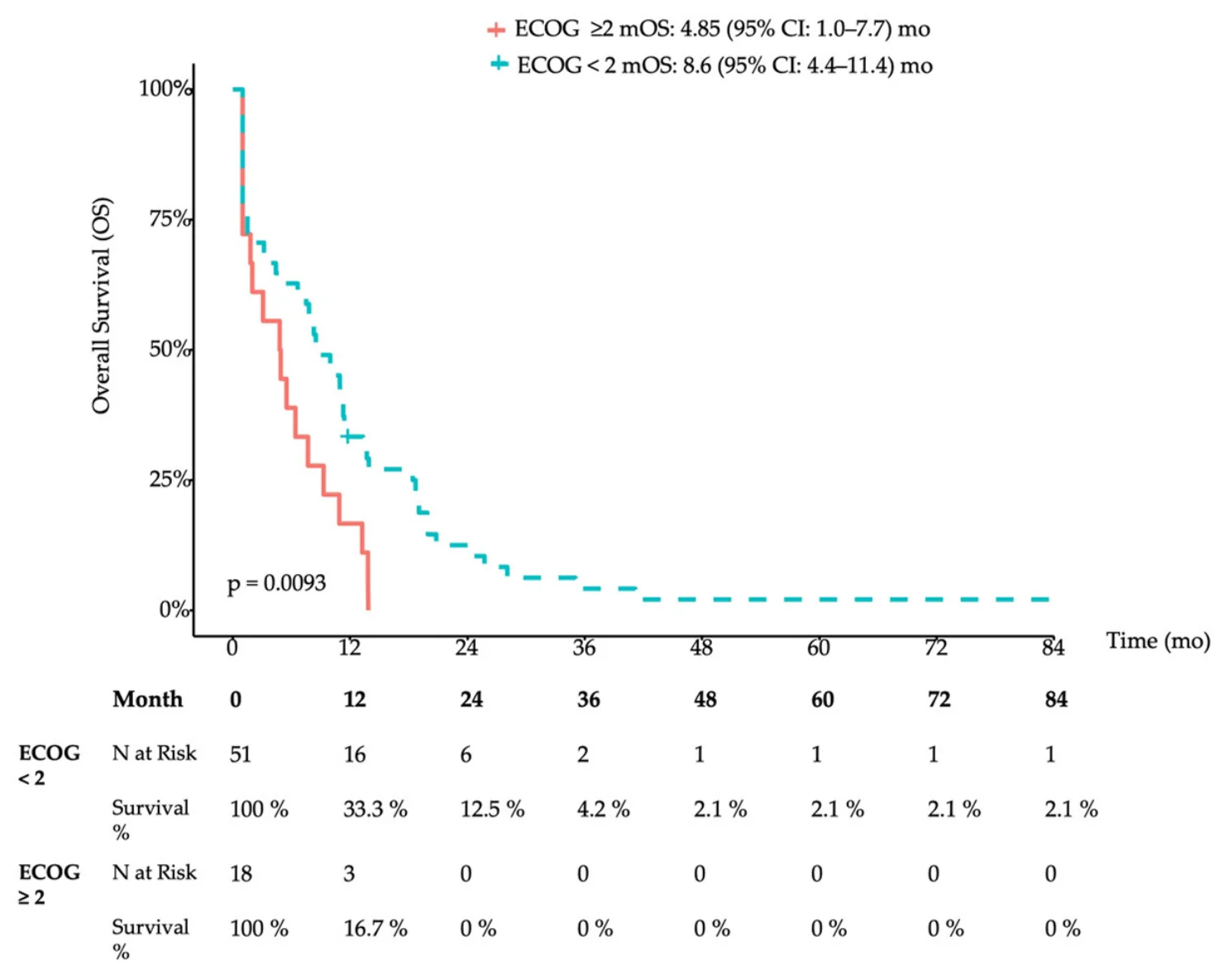
Kaplan–Meier overall survival curves stratified by ECOG performance status in older patients with acute myeloid leukemia (log-rank *p* = 0.009).

The median OS was 7.8 (range, 1–88.5) months and the 1-year and 2-year OS rates were 29% (95% CI: 20–41.9%) and 9.2% (95% CI: 4.3–19.5%), respectively. OS did not differ significantly according to sex, age, CCI, bone marrow blast number, peripheral blood blast percentage, leukocytosis or cytopenia, LDH level, eGFR, BMI or ELN classification. Patients with an ECOG performance status of ≥2 at the time of diagnosis had a statistically significantly shorter OS compared to those with better performance status (4.85 vs. 8.60 months, *p* = 0.009) (Figure 1).

### 3.3. Data of Patients with or Without Intensive Chemotherapy

Patients were categorized into intensive and non-intensive treatment groups. Among patients receiving intensive therapy, the median number of intermediate- or high-dose cytarabine consolidation cycles was 1 (range, 0–3). Patients receiving HMA monotherapy (azacitidine or decitabine) received a median of 4 treatment cycles (range, 1–20), whereas the three patients receiving azacitidine plus venetoclax received a median of 6 cycles (range, 2–13).

Baseline characteristics differed substantially between groups: intensive therapy was more frequently administered to patients younger than 70 years (*p* = 0.005), those with lower CCI scores (*p* < 0.001), and those with higher bone marrow (*p* = 0.011) and peripheral blood blast counts (*p* = 0.037). Leukocytosis was also more frequent among intensively treated patients (*p* = 0.004) (Table 3).

**Figure 2:**
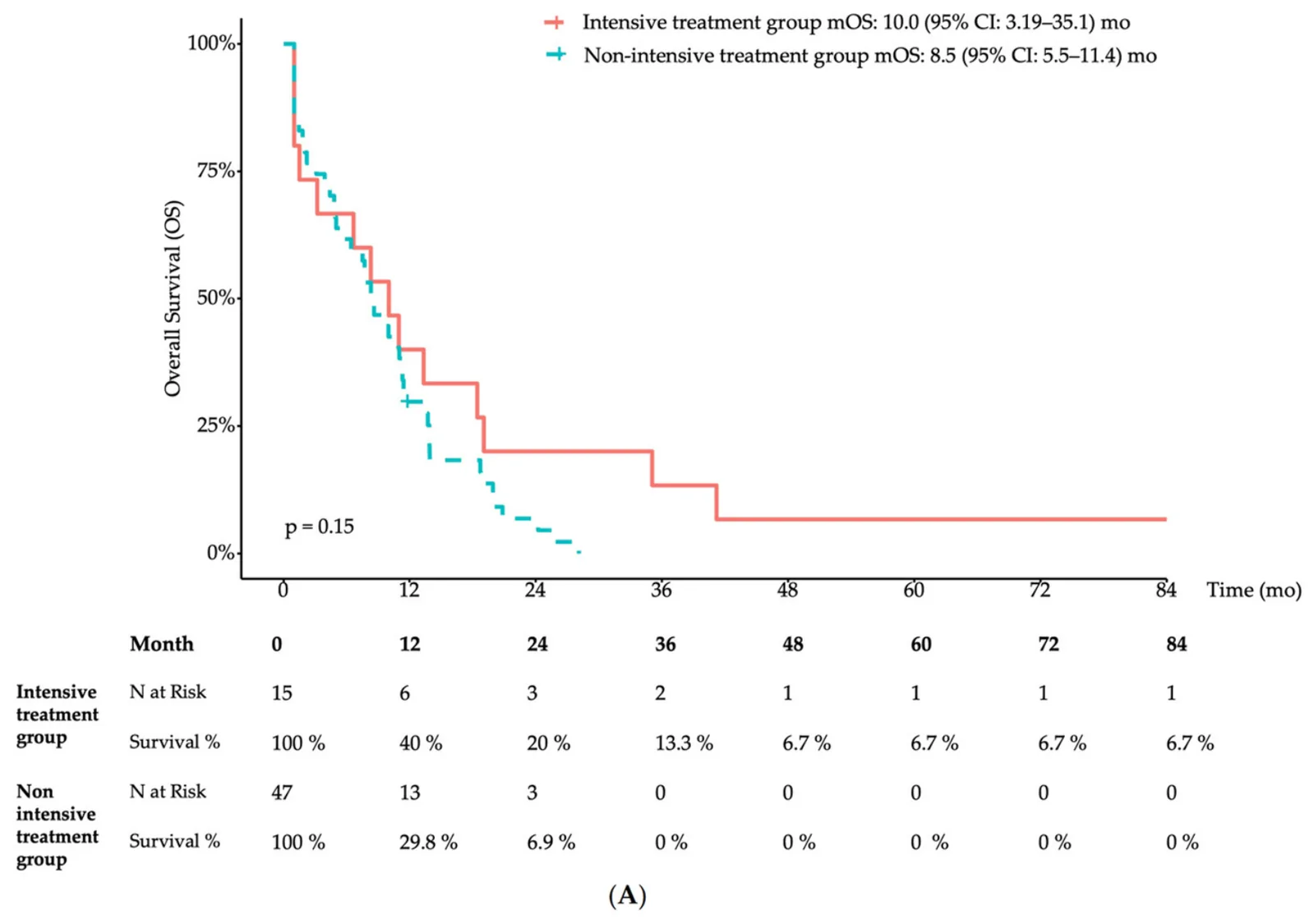

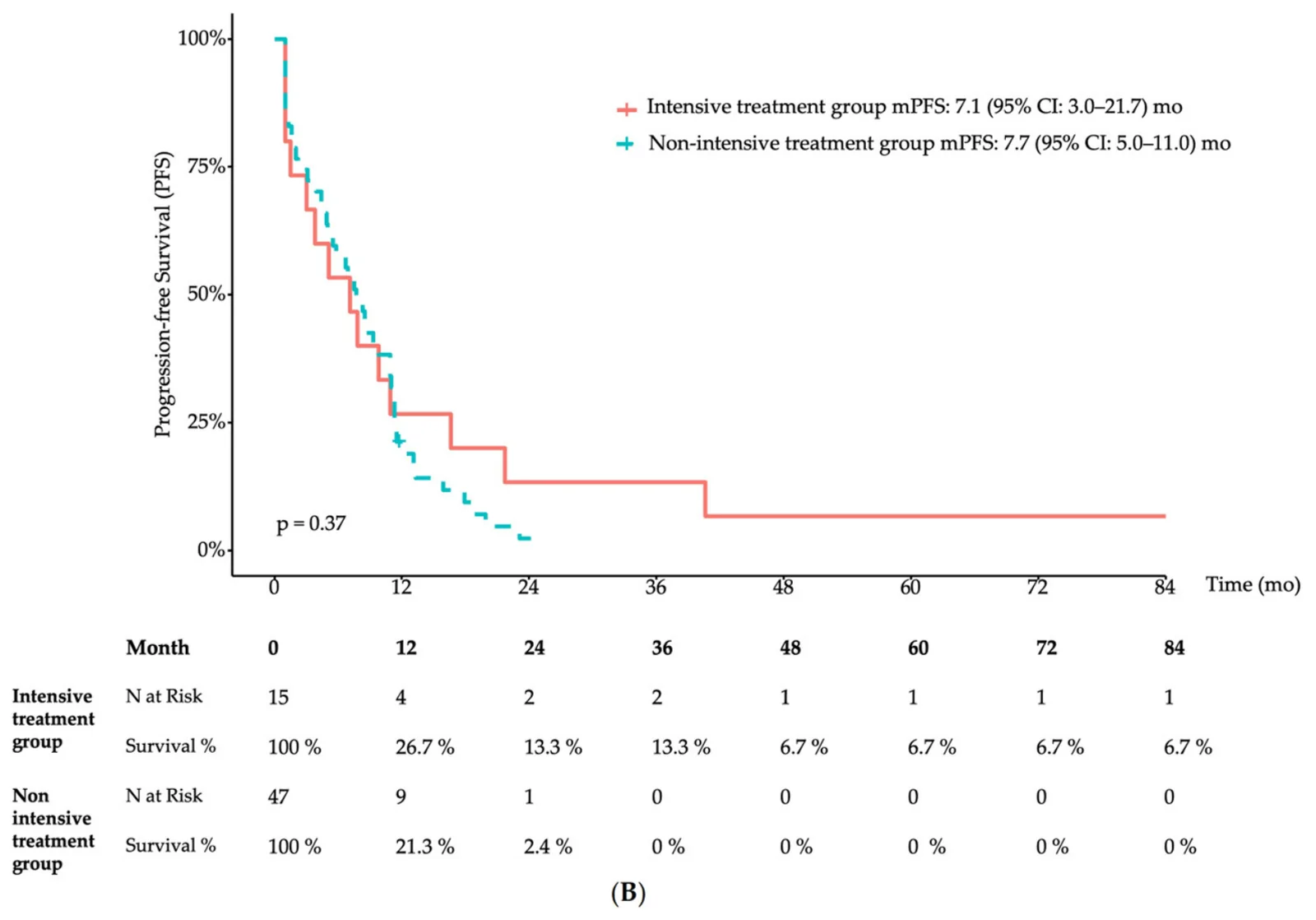
Kaplan–Meier overall survival (**A**) and progression-free survival (**B**) curves stratified by treatment intensity in older patients with acute myeloid leukemia. No significant differences were observed between the intensive and non-intensive treatment groups (overall survival, *p* = 0.15; progression-free survival, *p* = 0.37).

Among response-evaluable patients (n = 12 in the intensive group and n = 30 in the non-intensive group), CR/CRi was achieved in 7 patients (58.0%) receiving intensive therapy and 13 patients (43.3%) receiving HMA-based therapy; PR was observed in 2 (16.6%) and 11 (36.6%) patients, respectively. Response rates did not differ significantly between treatment groups. Median PFS was 7.1 months (95% CI: 3.0–21.7) in the intensive group and 7.7 months (95% CI: 5.0–11.0) in the non-intensive group (*p* = 0.370). Median OS was 10.0 months (95% CI: 3.19–35.1) and 8.5 months (95% CI: 5.5–11.4), respectively (*p* = 0.150) (Figure 2).

### 3.4. Safety

Antileukemic treatment could not be initiated in seven patients and safety data were reported in 62 patients. Treatment-related adverse events (AEs) were common in both groups, with a higher frequency and severity observed in the intensive treatment group. Any-grade AEs occurred in all patients (100%) receiving intensive therapy, compared with 95.7% in the non-intensive group. Severe AEs (grade ≥ 3) were reported in 100% and 85.1% of patients, respectively, without any statistical significance (Table 4).

Hematologic toxicities represented the most frequent adverse events. In the intensive group, neutropenia, thrombocytopenia, anemia, and febrile neutropenia occurred in all patients and were uniformly grade ≥ 3. In the non-intensive group, any-grade neutropenia was observed in 78.7% of patients (grade ≥ 3: 63.8%), thrombocytopenia in 74.4% (grade ≥ 3: 44.6%), anemia in 68% (grade ≥ 3: 36.2%), and febrile neutropenia in 38.3%. Grade ≥ 3 thrombocytopenia, anemia and febrile neutropenia were more common in the intensive therapy group with statistical significance (*p* = 0.031, *p* = 0.021 and *p* = 0.022, respectively).

Non-hematologic toxicities were also more prevalent in the intensive group. Infections were reported in all patients receiving intensive therapy, with severe infections in 60.0%, whereas infections occurred in 44.6% of patients in the non-intensive group, with grade ≥ 3 events in 40.4%. The most common infectious complication was pneumonia, affecting 24 patients overall, followed by urinary tract infections in 5 patients and otitis in 2 patients. Mucositis and diarrhea were more frequent and severe in the intensive group. Hepatotoxicity, tumor lysis syndrome, and electrolyte imbalance were infrequent and predominantly low grade. Among non-hematological toxicities, infections, mucositis, diarrhea, and electrolyte imbalance were significantly less frequent in the non-intensive treatment group (*p* = 0.033, *p* = 0.012, *p* = 0.028, *p* = 0.012, respectively).

## 4. Discussion

Older patients with AML pose a therapeutic challenge, as the role of chemotherapy-based induction remains controversial in this population. Although novel targeted therapies provide promising alternatives, limited accessibility and delayed availability—particularly in developing countries—often necessitate continued reliance on conventional induction approaches. Nevertheless, intensive chemotherapy may still represent a viable option for carefully selected, fit older patients [7,8]. In this context, real-world data remain important for understanding treatment patterns and outcomes across heterogeneous older AML populations.

In this retrospective real-world study of patients aged ≥ 65 years with AML, no statistically significant differences in progression-free or overall survival were observed between intensive and HMA-based treatment groups. Intensive chemotherapy was preferentially administered to younger patients with lower comorbidity burden and higher blast counts; however this selection did not translate into a statistically significant survival advantage. In contrast, ECOG performance status at diagnosis emerged as the only baseline factor significantly associated with overall survival, whereas age, comorbidity burden, laboratory parameters, and ELN risk classification were not associated with survival. As expected, treatment-related toxicities were more frequent in patients receiving intensive chemotherapy.

Treatment allocation in our cohort was strongly influenced by baseline patient characteristics. Non-intensive regimens were more frequently selected for older patients and for those with higher CCI scores. In particular, the use of intensive therapy was infrequent among patients aged ≥ 70 years and those with a CCI ≥ 6, and this difference was statistically significant. This preferential use of non-intensive therapy in older and more comorbid patients was consistent with real-world data demonstrating that treatment decisions in this population were strongly influenced by age, performance status, and comorbidities [9,10]. These findings reflect the importance of assessing patient fitness beyond chronological age alone when considering treatment intensity.

Treatment selection, however, appeared to be influenced not only by patient-related factors but also by disease burden. Patients receiving intensive therapy had higher bone marrow and peripheral blood blast percentages and were more likely to present with leukocytosis. This pattern may reflect the perceived need for more rapid cytoreduction in patients with a higher or more proliferative disease burden, particularly when intensive treatment was considered feasible based on age and comorbidity. Thus, treatment selection in our cohort appeared to reflect a balance between patient fitness and disease-related characteristics rather than treatment intensity being determined by either factor alone. Current American Society of Hematology (ASH) guidelines similarly emphasize an individualized approach, recognizing both conventional induction and HMA- or low-dose cytarabine (LDAC) -based therapy combined with venetoclax as treatment options for older patients considered candidates for intensive therapy. Conventional induction may be favored in younger older adults and those with favorable-risk disease, whereas venetoclax-based approaches may be preferred in patients aged ≥ 70 years or with certain poor-risk cytogenetic or molecular features [11]. Our study largely preceded the widespread adoption of venetoclax-based regimens, while limited comprehensive cytogenetic and molecular profiling restricted our ability to evaluate the influence of disease-risk features on treatment selection.

ELN risk stratification is one of the most important prognostic tools in AML and is known to become increasingly unfavorable with advancing age. Appelbaum et al. reported high-risk cytogenetic profiles in 35% of patients aged ≤ 55 years and 51% of those aged ≥ 75 years [12], while DiNardo et al. reported an adverse-risk rate of 49% among patients aged ≥ 65 years [13]. In our cohort, ELN risk classification could be performed in 52 patients (75.4%). Among classified patients, 23.1% had adverse-risk disease, which was lower than previously reported in the literature. This discrepancy should be interpreted with caution, as ELN risk stratification was feasible in only 75.4% of cases. Patients lacking risk classification were predominantly older and included those who experienced rapid clinical deterioration and early death before complete cytogenetic and molecular risk assessment. Therefore, adverse-risk disease may have been underrepresented in our cohort.

Among response-evaluable patients, 47.6% of patients achieved CR/CRi, while 31.0% attained PR. According to treatment intensity, CR/CRi was achieved in 58.0% of patients receiving intensive therapy and in 43.3% of those treated with HMA-based therapy, whereas PR was more frequently observed in the HMA group (36.6% vs. 16.6%). Although CR/CRi was numerically more frequent with intensive therapy in our cohort, no statistically significant difference in response rates was detected between treatment groups. This distribution may partly reflect differences in response kinetics, as intensive chemotherapy aims to achieve rapid cytoreduction and complete remission, whereas responses to HMA monotherapy may develop gradually and deepen over several treatment cycles [14]. In our cohort, patients treated with HMA monotherapy received a median of four cycles, which may have limited the time available to achieve deeper responses. Although the addition of venetoclax has substantially improved remission rates with HMA-based therapy, only three patients in our cohort received azacitidine plus venetoclax; therefore, our non-intensive treatment outcomes predominantly reflect HMA monotherapy rather than contemporary venetoclax-based therapy [5].

Beyond treatment type, CR/CRi rates did not differ significantly according to age, sex, ECOG performance status, CCI, LDH, eGFR, leukocytosis, or baseline cytopenias. However, the absence of significant associations should be interpreted cautiously, as treatment response in AML reflects the interplay between patient-related factors and disease biology. Kantarjian et al. demonstrated this heterogeneity among older patients receiving intensive chemotherapy, reporting CR rates exceeding 60% in patients with none or only one adverse prognostic factor, compared with less than 20% in those with three or more adverse factors, including advanced age, unfavorable karyotype, poor performance status or organ dysfunction, and longer duration of antecedent hematologic disease [15]. In particular, adverse or complex cytogenetic profiles and specific high-risk molecular alterations are well-established determinants of treatment resistance and lower likelihood of remission [16,17]. In our study, the relatively small number of response-evaluable patients and incomplete comprehensive molecular and cytogenetic profiling may have limited our ability to identify such predictors of response.

Despite the observed response rates, durable disease control remained challenging. In the ECOG-ACRIN trials including 3012 patients with newly diagnosed AML, 58.9% of patients who achieved first complete remission subsequently relapsed [18]. Similarly, disease progression occurred in 63.6% of patients who achieved at least a partial response in our cohort, emphasizing that an initial morphologic response does not necessarily translate into durable disease control. Morphologic remission alone may not fully capture the depth of treatment response, as measurable residual disease (MRD) provides additional prognostic information beyond conventional response assessment. Persistent MRD despite morphologic remission is associated with a higher risk of relapse and inferior survival [19,20]. MRD assessment was not routinely incorporated into clinical practice throughout the study period, precluding a consistent evaluation of its relationship with subsequent disease progression.

Consistent with the frequent progression observed after response, the short median PFS (6.7 months) and OS (7.8 months) indicate that initial responses often did not translate into durable disease control. The relatively narrow interval between PFS and OS may reflect the challenges of achieving effective disease control after progression in older patients with AML, given the limited efficacy and tolerability of salvage approaches reported in this population [21]. One- and two-year OS rates of 29% and 9.2%, respectively, further indicate that durable long-term survival was achieved in only a small proportion of patients. When analyzed according to treatment intensity, median OS was numerically longer with intensive therapy (10.0 vs. 8.5 months), whereas median PFS was similar between the intensive and non-intensive groups (7.1 vs. 7.7 months); neither difference reached statistical significance. However, these findings should not be interpreted as evidence of therapeutic equivalence between the two approaches.

The absence of a clear survival advantage for either treatment strategy in our cohort should be considered within the broader context of the heterogeneous evidence in older AML. Quintás-Cardama et al. reported similar survival with intensive chemotherapy and HMA-based therapy in 671 patients aged ≥ 65 years, despite higher remission rates with intensive treatment, while Vachhani et al. found no statistically significant difference in OS between the two approaches [10,22]. In contrast, a systematic review by Chang et al. suggested a modest survival advantage with intensive therapy, although the certainty of evidence was limited by confounding in the predominantly observational studies [23]. Variability across studies likely reflects differences in patient selection and fitness, cytogenetic and molecular risk profiles, treatment era, and the composition of less-intensive regimens. In particular, earlier cohorts predominantly evaluated HMA monotherapy, whereas the introduction of venetoclax-based combinations has substantially changed the efficacy of non-intensive treatment [5,24]. Differences in supportive care and treatment-related mortality may further contribute to variation in survival outcomes across real-world cohorts.

Among the baseline variables evaluated, OS was not significantly associated with baseline clinical, laboratory, or treatment-related variables, except for ECOG performance status, with patients having ECOG ≥ 2 experiencing significantly shorter OS. The adverse impact of ECOG performance status on survival has also been demonstrated in previous studies [15,24,25]. Notably, ECOG performance status reflects a dimension of patient fitness not fully captured by chronological age or comorbidity burden alone, including functional reserve, treatment tolerance, and overall vulnerability. Its stronger association with OS than with PFS in our cohort may therefore reflect its broader prognostic relevance beyond disease control alone. Nevertheless, prognosis in older AML remains multifactorial, with age, comorbidity burden, and disease-related characteristics also contributing to survival across different cohorts [25,26]. These findings support considering functional status alongside age, comorbidities, and disease biology when assessing prognosis and treatment suitability in older patients with AML.

Treatment-related toxicities were frequent in both groups but were more pronounced with intensive chemotherapy, particularly for hematologic and infectious complications. This finding is consistent with the recognized myelosuppressive burden of intensive induction therapy in older patients with AML [17]. Beyond conventional clinical predictors, emerging evidence suggests that host-related biological factors, including gut microbiome composition and treatment-related dysbiosis, may influence mucosal barrier integrity, susceptibility to infection, treatment toxicity, and potentially therapeutic response in AML [27]. These factors were not assessed in our cohort but may represent an additional source of interpatient variability in treatment tolerance and response.

### Limitations

This study has several limitations that should be acknowledged. First, its retrospective design inherently carries the risk of selection bias and missing data, which may have influenced both treatment allocation and outcome assessment. Survival comparisons were unadjusted, and the small sample size and incomplete molecular-risk data precluded reliable multivariable or propensity-based analyses. Second, the single-center nature of the study limits the generalizability of the findings. Molecular and cytogenetic risk stratification could not be performed in all patients, particularly in those who experienced early mortality, potentially leading to an underrepresentation of adverse-risk disease. Furthermore, treatment response was evaluable in only 42 patients, as 27 patients died before treatment initiation or formal response assessment, which may have introduced additional bias in the estimation of response rates. In addition, the non-intensive treatment group was heterogeneous, and only three patients received azacitidine plus venetoclax, limiting regimen-specific analysis. Finally, the long study period may also have introduced temporal heterogeneity due to changes in molecular testing, supportive care, and available treatment options, while adverse events may have been underreported because of the retrospective nature of data collection. Despite these limitations, this study provides useful real-world information on treatment patterns and outcomes in older patients with AML; however, the findings should be considered exploratory rather than evidence of therapeutic equivalence between treatment strategies.

## 5. Conclusions

In conclusion, no statistically significant differences in PFS or OS were observed between intensive and non-intensive treatment strategies in this real-world cohort of older patients with AML. Although CR/CRi rates were numerically higher with intensive therapy, relapse remained frequent among patients who achieved a response. ECOG performance status was significantly associated with overall survival, highlighting the prognostic relevance of functional status in this population. Treatment allocation was influenced by patient characteristics and disease burden, emphasizing the need for individualized treatment selection. Taken together, these findings provide real-world insight into the outcomes of different treatment strategies in a clinically heterogeneous older AML population; however, given the retrospective design and baseline differences between treatment groups, the observed absence of significant survival differences should not be interpreted as evidence of therapeutic equivalence.

## Figures and Tables

**Table 1:** Baseline Demographic and Clinical Characteristics.

Characteristic	
Age, median (range)	71 (65–88)
Sex (n, %)	
Female	31 (44.9%)
Male	38 (55.1%)
Subtype of AML (n, %)	
Primary (De novo) AML	52 (75.4%)
Secondary AML	17 (24.6%)
Myeloproliferative disease	4 (23.5%)
Chronic myelomonocytic leukemia	3 (17.6%)
Myelodysplastic syndrome	10 (58.9%)
ECOG performance status	
Median (range)	1 (0–3)
≥2 (n, %)	18 (26.1%)
<2 (n, %)	51 (73.9%)
Charlson Comorbidity Index (CCI)	
Median (range)	6 (4–9)
≥6 (n, %)	35 (50.7%)
≤5 (n, %)	34 (49.3%)
Comorbidities (n, %)	
Hypertension	32 (46.4%)
Diabetes mellitus	21 (30.4%)
Coronary artery disease	14 (20.2%)
Pulmonary disease	9 (13%)
Chronic renal failure	5 (7.2%)
Body mass index (median, range)	25 (18–34)

Abbreviations: AML, acute myeloid leukemia; CCI, Charlson Comorbidity Index; ECOG, Eastern Cooperative Oncology Group; MDS, myelodysplastic syndrome.

**Table 2:** Laboratory Tests at the Diagnosis.

Hemoglobin (g/L), Median (Range)	7.4 (4.1–11.8)
Leukocyte (×10^9^/L) (median, range)	7 (0.5–293)
≥50,000 (n, %)	14 (20.3%)
<50,000 (n, %)	55 (79.7%)
Platelets (×10^9^/L) (median, range)	53 (5–760)
LDH (IU/L) (median, range)	384 (134–2632)
High (n, %)	52 (75.4%)
eGFR (mL/min/1.73 m^2^) (median, range)	81 (18–108)
≤30 (n, %)	5 (7.2%)
30–50 (n, %)	8 (11.6%)
≥50 (n, %)	56 (81.2%)
Peripheral blood blast (%) (median, range)	24 (0–92)
Bone marrow blast (%) (median, range)	35 (20–98)

Abbreviations: eGFR, estimated glomerular filtration rate; LDH, lactate dehydrogenase.

**Table 3:** Comparison of Intensive and Non-Intensive Treatment Groups.

	Intensive Treatment Group (n = 15) n (%)	Non-intensive Treatment Group (n = 47) n (%)	*p* Value
Sex			0.184
Female	9 (34.6%)	17 (65.4%)	
Male	6 (16.7%)	30 (83.3%)	
Age, years			0.005
65–70	12 (42.9%)	16 (57.1%)	
>70	3 (8.8%)	31 (91.2%)	
Subtype of AML			0.739
Primary AML	12 (26.1%)	34 (73.9%)	
Secondary AML	3 (18.8%)	13 (76.5%)	
CCI			<0.001
≤5	14 (45.2%)	17 (54.8%)	
≥6	1 (3.2%)	30 (96.8%)	
Bone marrow blasts, %			0.011
≤50	4 (11.1%)	32 (88.9%)	
>50	11 (42.3%)	15 (57.7%)	
Peripheral blood blasts, % *			0.037
≤15	2 (7.1%)	26 (92.9%)	
>15	9 (33.3%)	18 (66.7%)	
Leukocytosis			0.004
Yes	11 (44.0%)	14 (56.0%)	
No	4 (8.5%)	33 (91.5%)	
Leukocyte count, ×10^9^/L			0.237
≥50	4 (40.0%)	6 (60.0%)	
<50	11 (21.2%)	41 (78.8%)	
Leukopenia			0.070
Yes	3 (11.1%)	24 (88.9%)	
No	12 (34.3%)	23 (65.7%)	
LDH			0.528
Normal	3 (17.6%)	14 (82.4%)	
High level	12 (26.7%)	33 (73.3%)	
eGFR, mL/min/1.73 m^2^			0.713
≤30	0 (0.0%)	4 (100%)	
30–50	2 (28.6%)	5 (71.4%)	
≥50	13 (25.5%)	38 (74.5%)	

Abbreviations: AML, acute myeloid leukemia; CCI, Charlson Comorbidity Index; eGFR, estimated glomerular filtration rate; LDH, lactate dehydrogenase. * Only patients with available data were included in the analysis.

**Table 4:** Adverse Events in Treatment Groups.

	Intensive Treatment Group (n = 15)		Non-Intensive Treatment Group (n = 47)	
	Any Grade, n (%)	Severe, n (%)	Any Grade, n (%)	Severe, n (%)
Adverse event	15 (100)	15 (100)	45 (95.7)	40 (85.1)
Hematologic adverse events				
Neutropenia	15 (100)	15 (100)	37 (78.7)	30 (63.8)
Thrombocytopenia	15 (100)	15 (100)	35 (74.4)	21(44.6)
Anemia	15 (100)	15 (100)	32(68)	17(36.2)
Febrile neutropenia	15 (100)	15 (100)	-	18 (38.3)
Non–hematologic adverse events				
Infection	15 (100)	9 (60)	21 (44.6)	19 (40.4)
Mucositis	15 (100)	6 (40)	7 (14.9)	0 (0)
Diarrhea	9 (60)	4 (26.6)	10(21.2)	2 (4.2)
Hepatotoxicity	3 (20)	1 (6.6)	10 (21.2)	3 (6.3)
Tumor lysis syndrome	1 (6.6)	0 (0)	2 (4.2)	0 (0)
Electrolyte imbalance	15 (100)	5 (33.3)	7(14.9)	0 (0)

Note: Severe adverse events were defined as grade 3 or higher.

## Data Availability

The data presented in this study are available from the corresponding author upon reasonable request. Data are not publicly available due to patient privacy and ethical restrictions.
